# Ortner’s Syndrome-A Rare Cause of Hoarseness: Its Importance to an Otorhinolaryngologist

**Published:** 2016-03

**Authors:** Vanita Sarin, Bhanu Bhardwaj

**Affiliations:** 1 *Department of Otorhinolaryngology, Sri Guru Ram Das Institute of Medical Sciences & Research, Amritsar, Punjab, India.*

**Keywords:** Cardiovocal, Hoarseness, Ortner’s syndrome.

## Abstract

**Introduction:**

Cardiovocal hoarseness (Ortner’s syndrome) is hoarseness of voice due to recurrent laryngeal nerve involvement secondary to cardiovascular disease. Recurrent laryngeal nerve in its course (especially the left side) follows a path that brings it in close proximity to numerous structures. These structures interfere with its function by pressure or by disruption of the nerve caused by disease invading the nerve. However painless asymptomatic intramural hematoma of the aortic arch, causing hoarseness as the only symptom, is a rare presentation as in this case.

**Case Report:**

We report a case of silent aortic intramural hematoma which manifested as hoarseness as the only presenting symptom. A detailed history and thorough clinical examination could not reveal the pathology of hoarseness. The cause of hoarseness was diagnosed as aortic intramural hematoma on contrast computed tomography. Thus the patient was diagnosed as case of cardiovocal hoarseness (Ortner’s syndrome) secondary to aortic intramural hematoma.

**Conclusion:**

A silent aortic intramural hematoma with hoarseness as the only presenting symptom is very rare. This particular case report holds lot of significance to an otolaryngologist as he should be aware of this entity and should always consider it in the differential diagnosis of hoarseness.

## Introduction

An intramural hematoma of the aortic arch is a rare cause of cardiovocal hoarseness also called as Ortner’s syndrome, first described in 1897 in patients with left atrial enlargement due to mitral valve stenosis (1).Though bronchogenic carcinoma is the commonest cause of extralaryngeal vocal fold palsy; the incidence of cardiovocal hoarseness cannot be overlooked.

Hoarseness due to cardiovascular cause is of utmost importance to an otolaryngologist as it is the indicator of impending life threatening complications and need early detection and active intervention at the right time. We report a case of silent aortic intramural hematoma which manifested as hoarseness as the only presenting symptom. The patient was medically managed by the department of cardiology and has been kept on regular follow up to evaluate the progression/regression of the disease process.

## Case Report

**Fig1:**
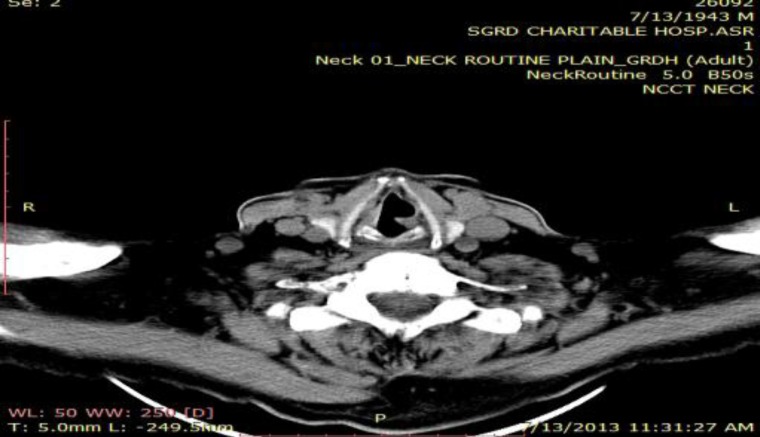
Contrast Enhanced computed tomography scan shows atrophy of the left thyroarytenoid muscle with enlargement of the left laryngeal ventricle indicating left laryngeal nerve palsy

A 70 year old male patient presented with the sole complaint of sudden onset of hoarseness of voice for the past 3 months. There was no history suggestive of any respiratory illness, sore throat, neck surgery, trauma, intubation, cardiovascular accident, exposure to any irritants, hypothyroidism, vocal abuse, valvular or ischemic heart disease. The patient was a non smoker, but a chronic alcoholic for the past 30 years. He was a known hypertensive for the past 5 years and was on irregular medication for the same. On clinical examination there was no stridor, goiter, cervical lymphadenopathy. His blood pressure was 140/90mmHg. His cardiovascular, respiratory and abdominal examinations were within normal limits. Indirect laryngoscopy and also a 90 rigid endoscopy revealed the left vocal fold fixed in paramedian position. Since no local cause of vocal fold palsy could be found, so further evaluation to find extralaryngeal causes of vocal fold palsy was done. Chest radiograph showed a left hilar opacity with mediastinal widening suggestive of a mediastinal mass. Laboratory investigations showed normal complete blood cell count. Serum lipid and thyroid levels were within normal limits. VDRL test was negative. Contrast enhanced computed tomography scans showed crescent shaped hypo attenuating area of size 6.8x1.8 cm along the left wall of aortic arch between the left common carotid and extending up to the left subclavian vessel with atherosclerotic plaque (Fig.1).

**Fig 2:**
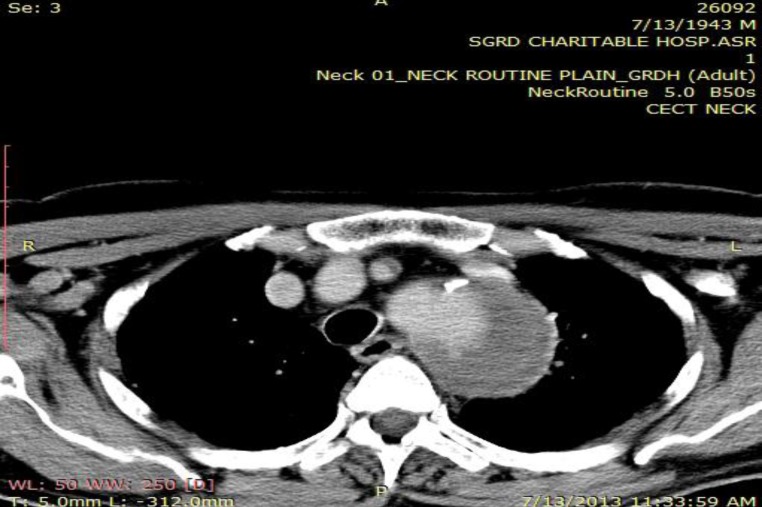
Contrast enhanced computed tomography scan shows crescent shaped hypo attenuating area of size 6.8x1.8 cm along the left wall of aortic arch between the left common carotid and extending up to the left subclavian vessel with atherosclerotic plaque

**Fig 3:**
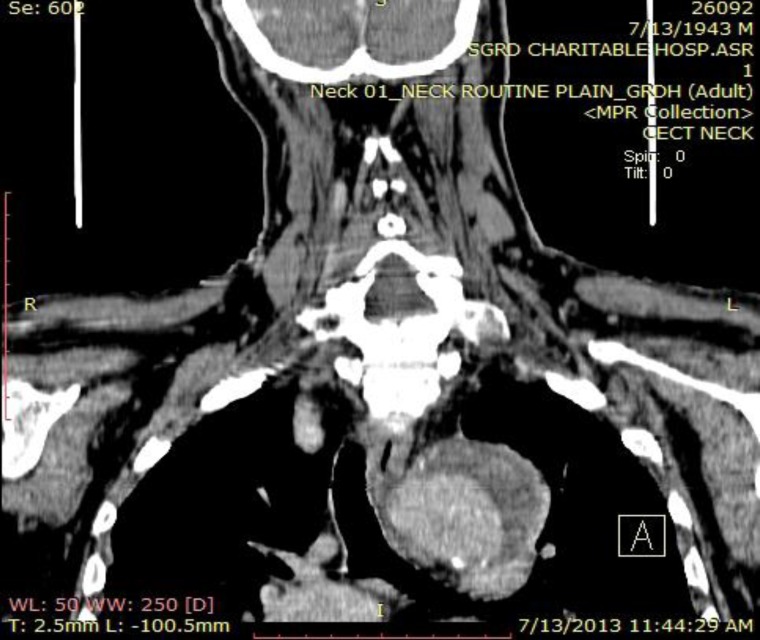
Contrast enhanced computed tomography scan shows crescent shaped hypo attenuating area of size 6.8x1.8 cm along the left wall of aortic arch between the left common carotid and extending up to the left subclavian vessel with atherosclerotic plaque

There was atrophy of the left thyroary- tenoid muscle with enlargement of the left laryngeal ventricle indicating left laryngeal nerve compression secondary to aortic intramural hematoma (Figs.2,3).

Even on retrospective repeated questioning, the patient did not divulge any other symptom regarding the disease except for hoarseness of voice. Patient was thus diagnosed as a case of cardiovocal hoarseness secondary to aortic intramural hematoma (Ortner’s syndrome). The patient was referred to the department of cardiology for further management.

## Discussion

Hoarseness of voice is a symptom resulting from interference of normal opposition of the vocal cords. It may be self limiting as in case of upper respiratory tract infections or vocal abuse. Common causes of vocal cord palsy in adults are malignant neoplasms (lungs, esophagus, thyroid, lymphoma and metastatic), trauma, surgery (thyroid, neck dissection, mediastinal), brain surgeries, inflammatory conditions like tuberculosis and idiopathic. However painless aortic intramural hematoma with hoarseness as the only symptom is a rare presentation, hence this case is reported.

The aortic wall consists of intima, media and adventitia with a thickness of 4mm. The intima is thin, the media contains elastic fibers and smooth muscle cells forming a spiral layer of tissue that provides the strength to the aortic wall and the adventitia provides the nutrition with the arterial and venous vasa vasorum. Aortic intramural hematoma often is described in literature as atypical aortic dissection because it is thought to represent either early stage limited dissection or thrombosis of the false lumen in dissection. Despite the similarity of its clinical manifestations and prognosis to those of both classic aortic dissection and penetrating aortic ulcer, intramural hematoma is considered as a distinct entity. The appearance of aortic wall hematoma without a demonstrable intimal flap was first described by Krukenberg in 1920 (2) and corresponds to a spontaneous rupture of the vasa vasorum followed by hemorrhage within the media and resultant weakening of the aortic wall (3). This disease entity is a matter of concern to the otorhinolaryngologists as it is associated with the mortality rate of 21% (4,6). Review of literature suggests that 94% of intramural hematomas had a non traumatic cause (5). The most common predisposing risk factor was hypertension seen in 53% of the cases with intramural hematomas. The signs and symptoms of this pathology may range from chest pain, back pain, syncope, anterior spinal syndrome, hoarseness, a diminished carotid pulse or acute renal insufficiency to variable electrocardiographic changes, aortic regurgitation and pericardial or pleural effusions (4,8). A silent aortic intramural hematoma with hoarseness as the only presenting symptom is very rare as is in this case.

Computed tomography is the preferred imaging modality for diagnosis, prognosis, regression and progression of the disease (9). Cross-sectional images show an enlarged overall aortic diameter, with or without compression of the aortic lumen. On unenhanced axial CT images, a crescentic, eccentric, hyper attenuating region of thickening of aortic wall (diameter>7mm) is considered diagnostic of acute intramural hematoma, in contrast to the multilayered pattern of increasing attenuation seen in aortic dissection, in which there is partial or complete thrombosis of the false lumen (4,10). In intramural hematoma as in aortic dissection, intimal calcifications may be displaced inward; however in presence of intramural hematoma such calcifications appear in semicircular or circular curvilinear configuration rather than linear configuration seen in presence of an intimal flap (4,5,10). On contrast enhanced axial CT images, the intramural fluid collection appears as a non-enhancing, smooth, crescentic region of aortic wall thickening that extends partially or entirely around the opacified aortic lumen, no spiraling of the intimal flap is seen (4,5,10,11). It’s important to document the maximal aortic diameter, the maximal axial thickness of the hematoma, and the minimum and maximum transverse diameters of the aortic lumen at the level of maximal intramural hematoma thickness. These characteristics are useful for predicting the outcome of an intramural hematoma. In addition, the absence of a dissection flap, intimal tear, or penetrating atherosclerotic ulcer is a pre-requisite for the diagnosis of intramural hematoma (5,7,12). 

However a number of complications may occur in the natural history of an intramural aortic hematoma that may require urgent intervention. A new intimal tear which is represented as an ulcer like projection from the site of the hematoma on axial CT images, may lead to aortic dissection. Moreover, saccular or fusiform aneurysmal dilatation of the aorta may occur at the site of the intramural hematoma and may lead to aortic rupture (7,12). For this reason, after an intramural hematoma is diagnosed, careful monitoring with regular follow up imaging is mandatory. Hoarseness due to vocal cord paralysis in patients with extra-laryngeal causes are common and may include very important diseases, most of which are life threatening, and a high index of suspicion is required to diagnose such situations. This particular case report holds lot of significance because though cardiovascular surgeons are well acquainted with this disease entity, it is one of the rare causes of hoarseness known to otorhinolaryngologists. Aortic intramural hematoma should be followed regularly because though some may resolve spontaneously, others pose a high risk of serious complications such as aortic dissection, aneurysm and rupture. Dissection may lead to additional complications such as aortic rupture, aortic regurgitation and cardiac tamponade. So appropriate clinical management is aided by accurate recognition of diagnostically specific CT features and awareness of their significance.

## Conclusion: 

A silent aortic intramural hematoma with hoarseness as the only presenting symptom is very rare. This particular case report holds lot of significance to an otolaryngologist as he should be aware of this entity and should always consider it in the differential diagnosis of hoarseness.
